# Fundamental and Practical Perspectives in Regenerative Medicine

**DOI:** 10.3390/ijms252111508

**Published:** 2024-10-26

**Authors:** Pavel I. Makarevich, Vsevolod A. Tkachuk

**Affiliations:** 1 Center for Regenerative Medicine, Medical Research and Education Institute, Lomonosov Moscow State University, 27-10 Lomonosovskiy Av., Moscow 119192, Russia; tkachuk@fbm.msu.ru; 2 Faculty of Medicine, Medical Research and Education Institute, Lomonosov Moscow State University, 27-1 Lomonosovskiy Av., Moscow 119192, Russia

## 1. Introduction and Problem Overview

As regenerative medicine continues to advance as a growing field in modern biology and the healthcare industry, it attracts enormous interest from the general public and scientists. The growth of this field is inevitably accompanied by numerous possible future directions, and regular revisions of current progress may be a helpful tool for both young and mature researchers. Increasingly complex technologies provide opportunities for cell fate manipulation (e.g., genome editing and reprogramming), tissue engineering, including “cell niche” recapitulation [1,2], and the application of stem-cell derived materials or drugs to control tissue repair and strike a balance between scarring and regeneration.

However, as we develop more tools to finely regulate regeneration processes, we continue to diverge from the natural course of events conventionally investigated in developmental biology, pathology and physiology which stood for centuries and yielded the basis that shaped the modern outlook of regenerative medicine [3]. Another issue that adds pressure to researchers concerns the application of regenerative medicine methods for human use as a clinical intervention which automatically encompasses one’s responsibility for its safety. Thus, the field of regenerative medicine is now standing at a point where we have to regularly update its progress and take time to re-evaluate the chosen directions and methodologies used so that we can provide fundamental knowledge and crucial derivatives of this type of medicine—new technologies and methods that can be translated into clinical use.

The collection of articles that we cover below provides a wide overview of the field, and we shall summarize the main future directions that may be of interest and were highlighted by the contributors.

## 2. Highlights of New Directions and Future Outreach

### 2.1. A General Outlook and Methodology in the Field

The crucial question that is stipulated by new-age researchers in the field is not “How can certain tissues regenerate and others cannot?”, but “How can we make our whole body capable of regeneration?”, which instantly provides two generally reasonable pathways. One involves searching for cases of regeneration in the human body (e.g., endometrium [4], distal phalanx regrowth [5]) and deconstructing them to find how these tissues are regulated and how they differ from other tissues which fail to re-grow in the same species. The other approach aims to identify and remove molecular and cellular blocks that make certain human tissues non-regenerative (typically, skin which heals by fibrosis). The common factor in these two paths is the *human,* whose physiology is in the spotlight rather than regenerating species which are evolutionarily distant from mammals and thus may possess mechanisms and reactions that are irreproducible in human medicine. Indeed, the majority of studies in regenerative medicine, including those published in this article collection, use human material along with relevant animal models applied to test an initial hypothesis or a method’s efficacy.

Generally, the modern outlook on regenerative medicine supports the concept of a *biomimetic approach,* which entails the application of natural mechanisms and targets the human body’s ability for renewal and regeneration. This direction includes the safety precautions that one should keep in mind once potential clinical application of a method is considered. It also serves as the pathway where fundamental discovery precedes potential use in treatment and new methods are merely instruments that we may apply with the necessary precaution and care.

Thus, despite significant translational success, one may not neglect the importance of fundamental research as the pivotal basis of new technologies in regenerative medicine. The wider use of transcriptome analysis and relevant animal models has become the marker of a new methodology which is less related to previous paradigms using *regenerating* species as a source of inspiration and tool for regenerative biology [6]. Our Special Issue comprising over 20 papers has illustrated the wide array of tools researchers use in this area of investigation—from machine analysis and patient-specific cell models to in vivo experiments accompanied by newly developed biomaterials and drug classes.

This methodology, along with new methods used to evaluate cell fate (e.g., lineage tracing), transcriptomic profile (single-cell RNA sequencing) and tissue structure (whole-mount microscopy and tissue clearing), will yield novel data on the course of events in normal and regenerating organs in mammals and—especially—humans.

Presenting a well-balanced view on tissue specificity and its relation to regenerative abilities provides a basis to merge developmental biology approaches with established methods in regenerative biology and physiology [7]. Basically, to understand the tissue-specific phenomena in adults, one has to deconstruct the course of tissue development in utero to elucidate how they are mediated and established. A crucial point for success is object choice, and the current article collection generously illustrates the range of objects that may be utilized for investigation.

### 2.2. Rise of Biomaterial Applications and Tissue Engineering

Another crucial change that has taken place in the application of stem cells is the shift from conventional cell therapy to tissue/organ engineering and the wide use of biomaterials. Such a change has been greatly inspired by progress in materials science which offers numerous chemical or natural compounds and polymers that mimic the environmental and signaling cues required for cells to survive and function after transplantation. Nevertheless, the main caveat that stands in the path of conventional cell therapy is the lack of efficacy due to cell death after injection-based delivery. This has hindered numerous clinical trials of stem cells, mesenchymal stromal cells (MSCs) and tissue-specific progenitors that were proposed for human use. Eventually, we hope to see a more “engineering-based” direction in regenerative medicine which relies on using multiple modalities to support cells ex vivo or give them properties that are deemed beneficial for their physiological and therapeutical potential [8]. This relates to numerous cell types—from adult MSCs to induced pluripotent stem cells (iPSCs)—that remain in the spotlight as potential tools for cell therapy and tissue engineering products. This direction is greatly supported by the conceptual point that cell functions are barely autonomous and highly depend on the niche environment which provides numerous signaling and mechanical cues [9,10,11].

Interestingly, with the extensive growth of iPSC-mediated models (especially for disease- or patient-specific cases), recently, a wide array of works has been published on the generation of immortalized cell lines from primary human material [12,13]. The spectrum of this research area is covered in a review by Voloshin et al. [14] which indicates the range from MSCs or adult stem cells to mature tissue elements [15,16], highlighting the efficacy of telomerase-driven immortalization for this task.

### 2.3. Cell-Free Methods Pave the Way for Translational Study

A blooming field in regenerative medicine that has recently gained attention is the application of *cell-free approaches* which involve the use of either in vivo *gene therapy* to influence physiological reactions and restore normal genotypes in hereditary ailments [17] or the application of cell *secretomes* for a wide spectrum of indications [18]. Cell secretomes obtained from cultures are considered a crucial factor that mediates the effects of cells delivered to damaged or dysfunctional tissues. Neither of the above-mentioned approaches require the presence of viable cells in the final formulation. Consequently, they possess the advantages of having a significant shelf-life and considerable safety, —the lack of which has limited the wider use of conventional cell therapy and tissue engineering.

Ironically, taking into account the history of regenerative medicine, one may recollect that after a tragic incident in gene therapy in 1999 [19], rising interest in cell therapy (including adult stem cells and MSCs) kept the field afloat. In the current situation where regenerative cell products are greatly staggered and overshadowed by the priority of anti-cancer cell-based methods, a great deal of success from vector-based and *ex vivo* gene therapies (including genetically modified stem cells) keeps driving the field toward translational milestones.

The current focus of gene therapy targets numerous hereditary ailments and conditions as it is the only reasonable etiotropic treatment for this class of diseases, which includes over 7,000 syndromes and diseases. However, once new targets are identified in regenerative biology, we can definitely expect new gene therapies that impact the corresponding processes and outcomes. The range of outcomes here is well established—reductions in fibrosis [20,21,22], the re-activation of regenerative capacity in postnatal tissues [23,24] and the regulation of neuro- [25,26]/angiogenesis, which are greatly related to healing outcomes in human [27,28]. Curiously, the frontier of gene therapy is highly related to the potential reprogramming of cell types in vivo, and one of the articles in this collection provides a detailed protocol (yet in vitro) for fibroblast-to-striated neuron transdifferentiation [29].

Targeting the secretome from human cells has become a new horizon dealing with the low survival rate of injected MSCs and adult progenitors, along with tissue engineering. While the latter focuses on harnessing the negative effects of tissue disruption by reconstructing the environment or its elements, therapies utilizing the secretome focus on the delivery of bioactive compounds secreted by cells in vitro [30]. This array of novel drugs utilizes molecules (or its fractions) isolated from cultured cells to directly impact the course of regenerative events in tissues or to regulate systemic conditions—from sepsis [31] to autoimmune diseases [30]. Once again, one may notice how the development of this field may turn the tide of the “bystander effect”. The latter is a term once used for the disappointingly obvious lack of incorporation of injected cells acting via the secretome until their death [32,33], and has become the basis of a novel class of biological medicinal products.

## 3. Conclusions

In recent decades, rapid change in regenerative medicine has become possible due to the peculiar convergence of fundamental investigations and the extensive development of new methods. These methods provide deeper insights into the molecular mechanisms of the phenomenon of regeneration and allow for the manipulation of cell fate, phenotype and tissue structure at an unprecedented level of rigor. Along with the legal and regulatory frameworks that have passed the stage of “trial and error” [34], these methods form the sustainable foundation of what we call regenerative medicine nowadays.

However, the prospects of this field will be conditionally divided into fundamental and practical as far as achievements in science are translated to treatment methods at a poorly predictable rate, with numerous obstacles. These should not divert attention from the everlasting process of knowledge gain, and such division is important to avoid “product-based” thinking among scientists.

Article collections, symposia and communication between scientists, physicians, regulators and patient communities are mandatory measures that enable us to apply the principle of individual expertise with a clear, set goal in sight. For regenerative medicine, translational achievements (clinical trials, marketed products) will become one of the heavyweight criteria for success—yet we should be aware of the pivotal role of fundamental investigators who set the basis for novel therapies and ideas.
